# Association between Fasting Glucose Concentration, Lipid Profile and 25(OH)D Status in Children Aged 9–11

**DOI:** 10.3390/nu10101359

**Published:** 2018-09-22

**Authors:** Lukasz Szternel, Magdalena Krintus, Katarzyna Bergmann, Tadeusz Derezinski, Grazyna Sypniewska

**Affiliations:** 1 Department of Laboratory Medicine, Nicolaus Copernicus University, Collegium Medicum, 85094 Bydgoszcz, Poland; krintus@wp.pl (M.K.); bergmann@vp.pl (K.B.); grazynaodes@interia.pl (G.S.); 2 Outpatient Clinic, “Esculap”, 88140 Gniewkowo, Poland; tadziude@wp.pl

**Keywords:** 25(OH)D deficiency, children, impaired fasting glucose, hypercholesterolemia

## Abstract

Background: The aim of this study was to assess the relationship between vitamin D status and the prevalence of dyslipidemia and impaired fasting glucose (IFG) in children. Methods and Summary: 284 children (150 boys and 134 girls) aged 9–11 were included in the study. Children with deficient 25(OH)D (25-hydroxycholecalciferol) levels ≤20 ng/mL (50 nmol/L) were characterized by a more frequent occurrence of impaired fasting glucose (IFG) (Odd ratios (OR) = 1.966, 95% confidence interval (CI): 1.055–3.663; *p* = 0.033) when compared to children with 25(OH)D >20 ng/mL. Serum 25(OH)D with concentration lower by 1 ng/mL (2.5 nmol/L) was linked to higher fasting glucose (by 0.25 mg/dL, 0.013 mmol/L; *p* = 0.017), higher total cholesterol (TC) by almost 1 mg/dL (0.96 mg/dL, 0.25 mmol/L; *p* = 0.006) and higher high-density lipoprotein cholesterol (HDL-C) (by 0.57 mg/dL, 0.015 mmol/L; *p* < 0.001). Conclusion: 25(OH)D deficiency may negatively affect fasting glucose and total cholesterol concentration in children aged 9–11. Vitamin D-deficient children are twice as likely to develop prediabetes as reflected by impaired fasting glucose when compared to those with a 25(OH)D level above 20 ng/mL (50 nmol/L).

## 1. Introduction

The prevalence of obesity and vitamin D deficiency among children makes this population demographic especially vulnerable to the development of these two pervasive epidemics [1]. Research suggests an unhealthy diet coupled with a sedentary lifestyle has become the main causal factor affecting the development of obesity, which in turn is frequently accompanied by dyslipidemia. A significant amount of data supports the hypothesis that optimal vitamin D concentration is linked to a favorable lipid profile and has a positive impact on glucose homeostasis [2]. Numerous observational, epidemiological, and cross-sectional studies indicate an inverse correlation between the concentration of 25(OH)D (25-hydroxycholecalciferol) and the rate of conversion from a prediabetes state to fully symptomatic diabetes mellitus [3]. In the USA, the prevalence of prediabetes (or according to the World Health Organization (WHO) definition, “intermediate hyperglycemia”) among adolescents aged 12–19 reached 13.1% between 2005 and 2006 [4]. It is estimated that annually 5%–10% of adults convert from a prediabetes state to overt diabetes, despite the fact that reversion to normoglycemia is much more common in children and adolescents. There are hypotheses indicating potentially beneficial effects of vitamin D in preventing transformation to fully symptomatic diabetes mellitus [3]. There is no consistent agreement as to whether vitamin D deficiency causes lipid abnormalities, or if these are just a consequence of excess adipose tissue mass storing 25(OH)D molecules. The inflammatory process links obesity and insulin resistance to the consequences of improper glucose homeostasis [5]. Vitamin D, being a negative marker of inflammation, seems to be a trigger molecule in the development of fully symptomatic metabolic syndrome [6]. The effects of vitamin D on lipid and carbohydrate metabolism may only be fully exploited with strong evidence from clinical trials of vitamin D supplementation.

The aim of this study is to assess the cross-sectional relationship between the status of vitamin D and the indices of metabolic pathways of lipids and glucose in a pediatric population.

## 2. Methods

### 2.1. Characteristics of the Study Participants and the Panel of Laboratory Tests

This cross-sectional study involved 284 presumably healthy children aged 9–11. The recruitment and blood collection process took place between October and November 2015. The children were selected on the basis of age (9–11 years old) from four primary schools in the Kujawsko-Pomorskie region of Poland. The second inclusion criterion was a fasting state (a minimum of 8 h since last meal) before blood drawing. Whilst school nurses and specialists in internal medicine participated in the recruitment process, the general health of the child on the day of study was subjectively evaluated by their parents. Children with any underlying liver, kidney, or endocrine diseases, or who were receiving drugs that affected vitamin D levels were excluded from the study. Immediately following blood collection, the blood samples were transported to the laboratory and centrifuged. Serum was used for further laboratory analysis. Whole blood samples were collected for HbA1_c_ evaluation. Vitamin D status (total 25(OH)D concentration), lipid panel (total cholesterol (TC), triglycerides (TG), high-density lipoprotein cholesterol (HDL-C), low-density lipoprotein cholesterol (LDL-C)), C-reactive protein (CRP), and glucose status (fasting glucose concentration and glycated hemoglobin) were evaluated for all participants.

### 2.2. Laboratory and Anthropometric Measurements

Concentrations of total 25(OH)D were analyzed on an IDS-iSYS automated analyzer (Immunodiagnostic Systems Holdings PLC (Didcot Way, Boldon, UK)) using IDS-iSYS 25(OH)D^S^ chemiluminescence assay for the quantitative determination of 25-hydroxyvitamin D and other hydroxylated metabolites (24,25(OH)_2_D_3_) [7]. The percentage of 25(OH)D_3_ and25(OH)D_2_ cross-reactivity was 97% and 120%, respectively. Cross-reactivity with epimers (3-epi-25(OH)D_3_, 3-epi-25(OH)D_2_) did not exceed 1% [8]. The reportable range for IDS-iSYS 25(OH)D^S^ assay ranged between 7 and 125 ng/mL (18–313 nmol/L). The assay used for the determination of 25(OH)D was traceable to isotope dilution-liquid chromatography/tandem mass spectrometry. Within-run precision of the IDS-iSYS 25(OH)D^S^ assay was evaluated by modified protocol CLSI EP-5A2 (Clinical and Laboratory Standard Institute, Evaluation of Precision Performance of Quantitative Measurement Methods) and ranged between 4.3% and 6.4% [8].

Lipid parameters including TC, TG, LDL-C, and HDL-C, high-sensitivity C-reactive protein (hs-CRP) and glucose concentration were measured with the use of an ABX Pentra 400 analyzer (Horiba Medical, Montpellier, France). Glycated hemoglobin was analyzed on a D-10™ Hemoglobin analyzer (BIO-RAD Diagnostics, Dublin, Ireland). All measurements were performed on fasting blood samples. The children’s height and weight was measured before blood collection and body mass index (BMI) percentiles determined using an online BMI calculator based on the “OLAF” project [9].

### 2.3. Definitions of Decision Criteria for Study Participants

The participants were divided according to the ADA (American Diabetes Association) recommendations, where a prediabetes condition is recognized when fasting glucose concentration is between 100 mg/dL and 125 mg/dL (5.6–6.9 mmol/L). On this basis, 234 (82.4%) children had fasting glucose concentrations <100 mg/dL (<5.6 mmol/L) and 50 (17.6%) children were recognized as having impaired fasting glucose (IFG), with fasting glucose concentrations ≥100 mg/dL (≥5.6 mmol/L) [10].

Risk of dyslipidemia was assessed in accord with the currently accepted cut-off values for fasting lipids in children [11]. Vitamin D status represented by 25(OH)D concentration was also evaluated according to currently accepted recommendations [12].

### 2.4. Statistical Analysis

Statistical analyses were carried out using SPSS (Statistical Package for the Social Sciences version 20, Armonk, NY, USA) software. The significance level was established at *p* < 0.05. The significance of the differences were determined using the *χ*^2^ (chi squared) test, or the Fisher exact probability test for small groups. All quantitative variables, except for TG and CRP, showed near-normal distributions. TG and CRP variables were logarithmically normalized to allow for further regression analysis.

The Fisher exact probability test was used to present significant differences in the percentage of normal glucose concentration and hyperglycemia in three subgroups of vitamin D status. Considering that only a small group had optimal 25(OH)D concentration (*n* = 10), the analysis of variables was undertaken incorporating a dichotomous division into those with deficiency states of 25(OH)D ≤ 20 ng/dL (<50 nmol/L) and those with 25(OH)D > 20 ng/dL (>50 nmol/L), respectively. The linear regression analysis was tested to estimate the impact of 25(OH)D and selected variables on glucose and lipid parameters. The incorporation of hs-CRP in logistic and linear regression analysis was essential to exclude any potential bias arising from possible inflammation status. In the linear regression analysis, we presented only statistically important models. Moreover, the variables included in regression analysis were chosen based on results from Pearson correlation coefficients. Multivariate logistic regression was performed for two-category variables. Because all statistical analyses were carried out in traditional units, SI units are placed in brackets.

### 2.5. Compliance with Ethical Standards

The study was approved by the local ethics committee (Nicolaus Copernicus University Collegium Medicum in Bydgoszcz, Poland) in accord with the Helsinki declaration and proper ethical standards (REB 338/2015). Informed consent was obtained from the parents.

## 3. Results

Our study consisted of 284 children: 150 girls (52.8%) and 134 boys (47.2%) aged 9–11. Characteristics of the study population are shown in Table 1. 

Overweight and obesity levels were based on BMI percentiles according to age and sex, being 11.6% and 12.3% respectively. Vitamin D deficiency, estimated by total 25(OH)D concentration, was identified in 37.7% of the children, whereas its insufficiency was identified in 58.8%. Children with impaired fasting glucose (≥100 mg/dL; 5.6 mmol/L) constituted 17.6%, and an elevated HbA1_c_ value was seen in 11.1% (Table 1). The most commonly identified lipid abnormality was hypercholesterolemia, which was 49.7% for TC and 35.6% for LDL-C, respectively.

Our analysis revealed that 25(OH)D concentration affected glucose metabolism as reflected by changes in fasting glucose concentrations. As can be seen in Table 2, the risk of hyperglycemia (IFG) was significantly higher (*p* = 0.033) in children with 25(OH)D deficiency (≤20 ng/mL; 50 nmol/L) (OR = 1.966, 95% CI: 1.055–3.663).

**Figure 1:**
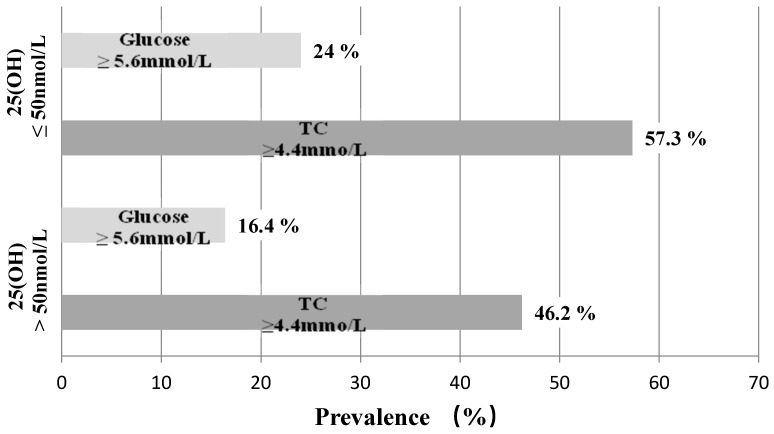
Occurrence of hyperglycemia/hypercholesterolemia in vitamin D-deficient children. TC: total cholesterol.

As can be seen in Figure 1, the prevalence of hyperglycemia and hypercholesterolemia was greater in the vitamin D deficiency subgroup (24.0% and 57.3%, respectively), when compared to children with concentrations of 25(OH)D over 50 nmol/L (20 ng/mL) (16.4% and 46.2%, respectively).

Pearson’s correlation coefficients across the whole study group revealed a statistically significant weak negative correlation between 25(OH)D level and lipid variables (TG: *r* = −0.160; *p* = 0.037; HDL-C: *r* = −0.209; *p* < 0.001), and also with glucose concentrations (*r* = −0.140; *p* = 0.018). A statistically significant correlation between 25(OH)D and glucose was identified in the hypercholesterolemia subgroup (*r* = −0.253; *p* = 0.002). A weak negative correlation (*r* = −0.215; *p* = 0.010) between 25(OH)D and TC was found in the normocholesterolemic subgroup (Table 3). A significant negative correlation of 25(OH)D with HDL-C was observed in children with hyperglycemia (*r* = −0.359; *p* = 0.010).

The linear regression model (Table 4) emphasized a significant correlation between 25(OH)D and glucose, and remained unchanged following adjustment for sex, age, and BMI percentiles.

Based on the first model presented, one can estimate that 25(OH)D reduced by 1 ng/mL (2.5 nmol/L) was related to slight, although significant, elevation of blood glucose concentration by 0.238 mg/dL (0.013 mmol/L). After adjustment for sex, age, and BMI, glucose concentration elevation was equal to 0.247 mg/dL (0.014 nmol/L) as a result of a unit decrease of 25 (OH)D concentration.

Our second model (Table 4) revealed a significant negative correlation between 25 (OH)D (*β* = −0.159; *p* = 0.006) and TC. The model presented showed a significant elevation in TC by 0.958 mg/dL (0.025 mmol/L) linked to a 25(OH)D reduction by 1 ng/mL (2.5 nmol/L). In the adjusted model, the TC elevation was identical.

The third linear regression model revealed an increase of HDL-C by 0.565 mg/dL (0.015 mmol/L), as a result of the 1 ng/mL (2.5 nmol/L) decrease in 25(OH)D concentration (*β* = −0.203; *p* < 0.001). Following adjustment, elevation of HDL-C reached 0.568 mg/dL (0.015 mmol/L) (Table 4).

## 4. Discussion

In the present study, the occurrence of IFG among children with vitamin D deficiency was almost two-fold higher (OR = 1.966) when compared to children with 25(OH)D higher than 20 ng/mL (50 nmol/L). We also observed a weak negative correlation between 25(OH)D and glucose concentration both in the whole group and in children with hypercholesterolemia.

We observed weak negative correlations between 25(OH)D and lipid components: TG and HDL-C in the whole group, HDL-C in the subgroup with hyperglycemia or TC and HDL-C in the subgroups with normo/hypercholesterolemia. Despite this weak correlation with lipid exponents, our study revealed a significant association between serum 25(OH)D and TC concentration. We found that serum 25(OH)D lower by 1 ng/mL (2.5 nmol/L) was linked to elevation in TC concentration by 0.96 mg /dL (0.025 mmol/L) and elevation of HDL-C by 0.57 mg/dL (0.015 mmol/L). The frequent occurrence of hypercholesterolemia in our study (57.3%), being a notable trend particularly in vitamin D-deficient children, is also worth noting. Evidence for the occurrence of dyslipidemia in relation to vitamin D status was inconsistent. It was observed that the concentration of TC was significantly higher in relation to vitamin D deficiency [13]. Our earlier study showed that HDL-C concentration was higher among 25(OH)D-deficient children with newly diagnosed asthma [14]. Other cross-sectional studies conducted independently by Reis et al. and Ashraf et al. did not find any significant association between low concentrations of vitamin D and abnormalities in lipid parameters [15,16]. A study by Jorde et al. revealed a cross-sectional relationship between 25(OH)D and serum lipids [17]. Meta-analysis conducted by Hao Wang et al. showed increased concentration of TC and LDL-C with simultaneously decreased concentrations of HDL-C and TG as influenced by vitamin D supplementation [18].

In this study we evaluated fasting glucose concentration, where abnormalities in this exponent of carbohydrate metabolism is considered as a first stage in prediabetes development. This condition may last several years without any additional symptoms, and depending on genetic or environmental background, may either revert to a normoglycemic state or develop into overt diabetes [19]. The prevalence of IFG among overweight and obese Hispanic children reported by Goran et al. ranged between 13% and 47% [20]. Our data indicated an inverse correlation between glucose and vitamin D concentrations, consistent with an earlier NHANES III (National Health and Nutrition Examination Survey) study where a negative relation between 25(OH)D and glycemia or diabetes occurrence was demonstrated [21]. In another study conducted on children and teenagers aged 12–19, it was seen that adjusted OR of fasting hyperglycemia among children with the lowest 25(OH)D (<15 ng/mL; 37 nmol/L) was two-and-a-half fold higher (2.54, 95% CI: 1.01–6.40) when compared to children in the highest 25(OH)D quartile (>26 ng/mL; 65 nmol/L) [15]. Our study showed that along with the decrease of 1 ng/mL (2.5 nmol/L) of 25(OH)D, serum concentration of fasting glucose increased by 0.25 mg/dL (0.014 mmol/L). The study conducted by Liu et al. indicated an association between 25(OH)D and diabetes, in contrast to that presented by Pittas et al., where it was suggested that there was insufficient data supporting hypotheses on the relation between vitamin D deficiency and its contribution to the risk of diabetes [22,23].

The most significant limitation of this study was sample size, which comprised over 100 dyslipidemic and only 50 hyperglycemic subjects. A further limitation of this study was its single-center and cross-sectional design which made it difficult to assess the influence of vitamin D on carbohydrate and lipid metabolism, with only the relationship between variables being tested. Therefore, our findings warrant confirmation in further studies. On the other hand, the strength of the present study was the simultaneous evaluation of two closely related metabolic pathways of glucose and lipids, in terms of the prevalence of vitamin D deficiency among children aged 9–11.

## 5. Conclusions

25(OH)D deficiency may negatively affect fasting glucose and total cholesterol concentration in children aged 9–11. Vitamin D-deficient children are twice as likely to develop prediabetes represented by IFG, when compared to those with 25(OH)D concentration above 20 ng/mL (50 nmol/L).

## Figures and Tables

**Table 1:** Characteristics of the study population (*n* = 284).

	Parameters	*n*	(%)	Mean (±SD) or Median (27–75th Percentile)	* *p* (<0.05)
**Age**	9 years	112	(39.4)	-	
	10 years	97	(34.2)	-	
	11 years	75	(26.4)	-	
**Sex**	Boys	150	(52.8)	-	
	Girls	134	(47.2)	-	
**BMI percentiles**	<5 (underweight)	16	(5.6)	26.8 (± 6.9)	
	≥5 and <85 (optimal weight)	200	(70.4)	34.4 (± 6.0)	<0.001 **
	≥85 and <95 (overweight)	33	(11.6)	46 (± 6.8)	
	≥95 (obese)	35	(12.3)	56.1 (± 12.1)	
**Glycemic status**	Glucose (<100 mg/dL; 5.6 mmol/L)	234	(82.4)	90 (± 6.5); 5.0 (± 0.36)	<0.001
	Glucose (≥100 mg/dL; 5.6 mmol/L)	50	(17.6)	105 (± 6.5); 5.8 (± 0.36)	
	HbA1_c_ (<5.7 %; 38 mmol/mol)	249	(88.9)	5.3 (± 0.2); 34.5 (±2.2)	
	HbA1_c_ (≥5.7 %; 38 mmol/mol)	31	(11.1)	5.8 (± 0.16); 40.0 (±1.7)	
**Lipids**	TC (<170 mg/dL; 4.4 mmol/L)	143	(50.3)	148 (± 14.1); 3.85 (± 0.37)	0.919
	TC (≥170 mg/dL; 4.4 mmol/L)	141	(49.7)	194 (± 22.0); 5.02 (± 0.57)	
	TG: 0–9 years (<75 mg/dL; 0.85 mmol/L)	67	(23.6)	53 (± 12.8); 0.60 (± 0.14)	<0.001
	TG: 10–19 years (<90 mg/dL; 1.02 mmol/L)	123	(43.3)	62 (± 15.8); 0.70 (± 0.18)	
	TG: 0–9 years (≥75 mg/dL; 0.85 mmol/L)	46	(16.2)	112 (± 39.0); 1.27 (± 0.44)	
	TG: 10–19 years (≥90 mg/dL; 1.02 mmol/L)	48	(16.9)	136 (± 42.6); 1.54 (± 0.48)	
	LDL-C (<110 mg/dL; 2.85 mmol/L)	183	(64.4)	86 (± 14.6); 2.23 (± 0.38)	<0.001
	LDL-C (≥110 mg/dL; 2.85 mmol/L)	101	(35.6)	129 (± 20.8); 3.34 (± 0.54)	
	HDL-C (>45 mg/dL; 1.17 mmol/L)	252	(88.7)	62 (± 11.2); 1.60 (± 0.29)	<0.001
	HDL-C (≤45 mg/dL; 1.17 mmol/L)	32	(11.3)	40 (± 4.4); 1.04 (± 0.11)	
	non-HDL-C (<120 mg/dL; mmol/L)	178	(62.7)	96 (± 15.3); 2.47 (± 0.40)	<0.001
	non-HDL-C (≥120 mg/dL; mmol/L)	106	(37.3)	139 (± 21.6); 3.59 (± 0.56)	
**hs-CRP**	(<1 mg/L)	196	(69.0)	0.2 (0.12–0.40)	<0.001
	(≥1 mg/L)	88	(31.0)	2.1 (1.5–3.8)	
**25(OH)D status**	Optimal (≥30 ng/mL; 75 nmol/L)	10	(3.5)	31.8 (± 1.5); 79.37 (± 3.74)	
	Insufficiency (21–29 ng/mL; 52–72 nmol/L)	167	(58.8)	23.7 (± 2.6); 59.16 (± 6.49)	0.007
	Deficiency (≤20 ng/mL; 50 nmol/L)	107	(37.7)	17.0 (± 2.3); 42.43 (± 5.74)	

* Difference between two structure indicators determined by Chi Square test. ** Optimal vs. overweight and obese. NA: not applicable. BMI: body mass index; HbA1_c_: glycated hemoglobin; TC: total cholesterol; TG: triglycerides; LDL-C: low-density lipoprotein cholesterol (direct method); HDL-C: high-density lipoprotein cholesterol; non-HDL-C: non-high-density lipoprotein cholesterol (TC-(HDL-C)); hs-CRP: high-sensitivity C-reactive protein; 25(OH)D: 25-hydroxycholecalciferol (calcifediol); *p*: statistical significance (<0.05). Unit conversion factors: glucose ((mg/dL) × 0.05551 = mmol/L); HbA1_c_ (10.93 × (HbA1_c_%) − 23.5 = mmol/mol); TC, HDL-C, LDL-C ((mg/dL) × 0.0259 = mmol/L); TG ((mg/dL) × 0.0113 = mmol/L); 25(OH)D ((ng/mL) × 2.496 = nmol/L).

**Table 2:** Association of 25(OH)D deficiency with other variables.

Variables	* *p* Value	OR	95% CI	
			Lower	Upper
Age	0.791	0.958	0.699	1.314
Sex	0.636	0.885	0.534	1.467
BMI percentiles	0.053	0.665	0.440	1.005
TC	0.169	1.688	0.800	3.562
TG	0.973	1.010	0.565	1.804
HDL-C	0.245	0.611	0.267	1.402
Non-HDL-C	0.624	1.267	0.493	3.257
LDL-C	0.257	0.599	0.247	1.454
hs-CRP	0.935	0.975	0.531	1.791
HBA1_C_	0.290	1.529	0.696	3.357
Glucose	0.069	1.828	0.955	3.499
Glucose **	0.033	1.966	1.055	3.663

Logistic regression (multivariate): *χ*^2^ = 13.5; df = 11 (degrees of freedom); *p* = 0.258; r2 Cox and Snell = 0.047 (Cox and Snell’s R squares complex samples logistic regression algorithms); r2 Nagelkerke = 0.064 (Nagelkerke R squares complex samples logistic regression algorithms) * Statistical significance was determined in logistic regression analysis. ** Logistic regression (univariate): *χ*^2^ = 4.513; df = 1; *p* = 0.034; r2 Cox and Snell=0.016; r2 Nagelkerke = 0.022. 25(OH)D: 25-hydroxycholecalciferol (calcifediol); BMI: body mass index; TC: total cholesterol; TG: triglycerides; HDL-C: high-density lipoprotein cholesterol; non-HDL-C: non-high-density lipoprotein cholesterol (TC-(HDL-C)); LDL-C: low-density lipoprotein cholesterol (direct method); hs-CRP: high-sensitivity C-reactive protein; HbA1_c_: glycated hemoglobin.

**Table 3:** Pearson’s correlation coefficients in normo-/hyperglycemic and normo-/hypercholesterolemic state.

Vitamin D/R-Pearson Correlation		TC	TG	HDL-C	Non-HDL-C	LDL-C	Glucose	HbA1_C_	hs-CRP
**Glucose: <100 mg/dL (<5.6 mmol/L) ((*n* = 234)—normoglycemia)**									
25(OH)D	(*R*) ^1^	−0.136	0.001	−0.163	−0.066	−0.064	−0.122	0.131	−0.001
	(*p*) ^2^	0.037	0.990	0.012	0.314	0.330	0.063	0.046	0.989
**Glucose: ≥100 mg/dL (≥5.6 mmol/L) ((*n* = 50)—hyperglycemia)**									
25(OH)D	(*R*) ^1^	−0.251	−0.025	−0.359	−0.066	−0.135	−0.087	−0.008	−0.041
	(*p*) ^2^	0.079	0.862	0.010	0.647	0.351	0.547	0.956	0.779
**Total cholesterol ≤170 mg/dL (≤4.4 mmol/L) ((*n* = 143)—normocholesterolemia)**									
25(OH)D	(*R*) ^1^	−0.215	−0.019	−0.187	−0.066	−0.035	−0.019	0.050	−0.053
	(*p*) ^2^	0.010	0.820	0.026	0.436	0.677	0.821	0.554	0.531
**Total cholesterol ≥170 mg/dL (≥ 4.4 mmol/L) ((*n* =141)—hypercholesterolemia)**									
25(OH)D	(*R*) ^1^	−0.030	0.039	−0.181	0.091	0.030	−0.253	0.154	0.051
	(*p*) ^2^	0.724	0.644	0.032	0.282	0.722	0.002	0.070	0.546
**All (*n* = 284)**									
25(OH)D	(*R*) ^1^	−0.005	−0.160	−0.209	−0.067	−0.078	−0.140	0.091	0.001
	(*p*) ^2^	0.932	0.007	<0.001	0.262	0.193	0.018	0.129	0.991

^1^*R*: Pearson correlation. ^2^ Statistical significance (*p* < 0.05), established using Pearson’s correlation analysis. 25(OH)D: 25-hydroxycholecalciferol (calcifediol); TC: total cholesterol; HDL-C: high-density lipoprotein cholesterol; non-HDL-C: non-high-density lipoprotein cholesterol (TC-(HDL-C)); LDL-C: low-density lipoprotein cholesterol (direct method); HbA1_c_: glycated hemoglobin; TG: triglycerides (logarithmic transformation); hs-CRP: high-sensitivity C-reactive protein (logarithmic transformation).

**Table 4:** Impact of 25(OH)D concentration on glucose and lipids.

Model	Independent Variables	Adjusted r^2^	β (beta)(Standardized coefficients)	B (Unstandardized Coefficients)	* *p* Value
**Model 1**		0.020			0.021
**Glucose**	25(OH)D			−0.238	0.021
	CRP log		−0.087	−1.058	0.139
Model 1 adj.		0.027			0.027
Glucose	25(OH)D		−0.142	−0.247	0.017
	CRP log		−0.107	−1.303	0.106
**Model 2**		0.052			<0.001
**TC**	25(OH)D		−0.159	−0.955	0.006
	TG log		0.183	0.124	0.002
Model 2 adj.		0.048			**0.002**
TC	25(OH)D		−0.160	−0.958	0.006
	TG log		0.192	0.130	0.003
**Model 3**		0.097			<0.001
**HDL-C**	25(OH)D		−0.202	−0.565	<0.001
	CRP log		−0.244	−4.769	<0.001
Model 3 adj.		0.093			<0.001
HDL-C	25(OH)D		−0.203	−0.568	<0.001
	CRP log		−0.249	−4.860	<0.001

* Statistical significance established using multiple linear regression analysis unadjusted and adjusted by sex, age, and BMI percentiles. Model adj.: model adjusted by sex, age, and BMI percentiles. 25(OH)D: 25-hydroxycholecalciferol (calcifediol); hs-CRP: high-sensitivity C-reactive protein (logarithmic transformation); TG: triglycerides (logarithmic transformation); TC: total cholesterol; HDL-C: high-density lipoprotein cholesterol.

## Abbreviations

25(OH)D	25-Hydroxycholecalciferol
ADA	American Diabetes Association
BMI	Body Mass Index
CLSI	Clinical and Laboratory Standards Institute
FG	Fasting Glucose
HbA1_c_	Glycated Hemoglobin
HDL-C	High Density Lipoprotein Cholesterol by direct method
HS-CRP	High Sensitivity C-Reactive Protein
IFG	Impaired Fasting Glucose
LDL-C	Low Density Lipoprotein Cholesterol by direct method
NHANES	National Health and Nutrition Examination Survey
TC	Total Cholesterol
TG	Triglycerides
WHO	World Health Organization
